# Efficacy and safety of omega-3 fatty acids on liver-related outcomes in patients with nonalcoholic fatty liver disease: A protocol for a systematic review and meta-analysis

**DOI:** 10.1097/MD.0000000000020624

**Published:** 2020-06-12

**Authors:** Xiao-yan Shi, Si-min Fan, Guo-mei Shi, Jia Yao, Yang Gao, Yu-guo Xia, Qiu Chen

**Affiliations:** a Hospital of Chengdu University of Traditional Chinese Medicine, Chengdu, Sichuan Province; b School of Information Science and Technology, Northeast Normal University, Changchun, Jilin Province, P.R. China.

**Keywords:** liver enzymes, liver histology, non-alcoholic fatty liver disease, omega-3 polyunsaturated fatty acids, protocol, systematic review

## Abstract

**Background:**

Non-alcoholic fatty liver disease (NAFLD), especially non-alcoholic steatohepatitis, which is considered as the hepatic manifestation of metabolic syndrome, has a great prevalence all over the world. New drugs are urgently needed for the treatment of NAFLD. This review will be to assess the efficacy and safety of omega-3 polyunsaturated fatty acids (n-3 PUFAs) on liver-related outcomes (liver histology and liver enzymes) in patients with NAFLD.

**Methods:**

We will search 5 databases for relative studies: Medline, the Cochrane Library, EMBASE, Web of Science, and ClinicalTrials.gov and identified all reports of randomized controlled trials published prior to July 2020. Two authors will independently scan the articles searched, extract the data from articles included, and assess the risk of bias by Cochrane tool of risk of bias. Disagreements will be resolved by discussion among authors. All analysis will be performed based on the Cochrane Handbook for Systematic Reviews of Interventions. Fixed-effects model or random-effects model will be used to calculate pooled estimates of weighted mean difference with 95% confidence intervals.

**Results:**

This systematic review aims to examine the effect of n-3 PUFAs on liver histology and liver enzymes in patients with NAFLD.

**Conclusions:**

These findings will provide guidance to clinicians and patients on the use of n-3 PUFAs for NAFLD.

**Ethics and dissemination:**

This study is a protocol for a systematic review of n-3 PUFAs as a treatment of NAFLD patients. This review will be published in a journal and disseminated in print by peer-review.

**Systematic review registration:**

INPLASY202050008.

## 1 Introduction

Nonalcoholic fatty liver disease (NAFLD) is becoming the commonest chronic liver disease, affecting up to 25% to 30% of the general population^[1]^ and 76% to 90% of patients with components of metabolic syndrome (MetS).^[1–3]^ Even its prevalence is thought to be rising as the unhealthy lifestyle and diet.^[4]^ The two pathological conditions of NAFLD include non-alcoholic fatty liver and non-alcoholic steatohepatitis (NASH) (with inflammation and ballooning, with or without fibrosis).^[5]^ The latter, the more severe form of NAFLD, can predict fibrosis progression. Up to 9% to 20% and 22% of NASH patients can progress to cirrhosis and die from liver-related severe sequelae, respectively.^[6]^ In the western countries, NAFLD, especially NASH, has been recognized as 1 of the leading causes of cirrhosis and liver transplantation and has been shown to be present in 59% of patients with hepatocellular carcinoma.^[7,8]^

So far, still no pharmacologic therapy is approved for NAFLD, reductions in body weight through lifestyle intervention remain the first-line therapy. However, most patients cannot achieve the required degree of weight loss and long-term weight control is difficult for them as well.^[9]^ In the last decade, the close relationship between NAFLD and omega-3 polyunsaturated fatty acids (n-3 PUFAs) metabolism has been growing interest. The low dietary intake of n-3 PUFAs was found in NAFLD patients and the high hepatic N-6: N-3 ratio in the liver may favor lipid synthesis, lead to steatosis and induce insulin resistance, thereby promoting the progress of NAFLD.^[10,11]^ Moreover, n-3 PUFAs for years has been known as potent modulators of hepatic gene expression. It could favor fatty acid oxidation and inhibit lipogenesis through diminishing sterol receptor element-binding protein-1 (SREBP1c) and increase peroxisome proliferator-activated receptor-a (PPAR-a).^[12]^ Animal studies have suggested that n-3 PUFAs can reduce hepatic steatosis and markers of inflammation, and improve metabolic parameters.^[13,14]^ In addition, the effects of n-3 PUFAs on reducing adiposity and altering body composition have been also reported.^[15]^

However, clinical trials investigating the efficacy of n-3 PUFAs supplements on NAFLD patients have reported inconsistent results. Three meta-analyses pooled results from randomized controlled trials (RCTs) and Non-randomized controlled trials (Non-RCTs) show that n-3 PUFAs treatment had a beneficial effect on liver fat in NAFLD patients than control patients, but yielded controversial effects on liver enzymes.^[16–18]^ And there is a lack of pooled results in liver histology. After those publications, many additional RCTs studying the effects of n-3 PUFAs therapy in NAFLD patients (especially in children) have been published. Given the uncertainties in the effects of n-3 PUFAs therapy on liver enzymes and liver histology among NAFLD patients, we conduct the present meta-analysis based on RCTs to assess the efficacy of n-3 PUFAs supplementation on liver-related outcomes and its safety in patients with NAFLD. And these findings may provide guidance to clinicians and patients on the use of n-3 PUFAs for NAFLD.

## 2 Methods

### 2.1 Registration

Our meta-analysis protocol was registered in the International platform of registered systematic review and meta-analysis protocols (INPLASY) as number INPLASY202050008.

This study will be designed in accordance with the Preferred Reporting Items for Systematic Reviews and Meta-analysis (PRISMA-P) guidelines.^[19]^

### 2.2 Eligibility criteria

We will include studies according to the following inclusion criteria:

(1) study design: RCTs with any follow-up duration and sample size were allowed;
(2) population: patients of any age or sex or ethnic origin with a definitive diagnosis of NAFLD or NASH by histologic or imaging evidence (ultrasound, computed tomography or magnetic resonance imaging (MRI);
(3) intervention: n-3 PUFAs at any dose and route;
(4) control: placebo, or other active agents;
(5) outcomes: primary outcomes are liver histology including steatosis score, hepatocellular ballooning score, lobular inflammation score, fibrosis score and NAFLD activity score (NAS).

Secondary outcomes are liver enzymes including alanine aminotransferase, aspartate transaminase, γ-glutamyl transferase, and adverse events.

### 2.3 Search methods for the identification of studies

Two authors (XS and SF) will independently search databases including Medline, the Cochrane Library, EMBASE, and Web of Science until July 2020. According to the PICOS principle, the keywords of our search terms were: (“eicosapentaenoic acid” OR “docosahexaenoic acid” OR “omega-3” OR “ω-3” OR “EPA” OR “DHA” OR “PUFAs” OR “n-3 PUFAs”) AND (“non-alcoholic fatty liver disease” OR “non alcoholic fatty liver disease” OR “NAFLD” OR “nonalcoholic fatty liver disease” OR “nonalcoholic fatty liver^∗^” OR “nonalcoholic steatohepatiti^∗^” OR “non-alcoholic steatohepatitis^∗^” OR “fatty liver^∗^” OR “NASH”). The ClinicalTrials.gov registry will also be searched for unpublished trials and the authors will be contacted for any additional information if necessary. Relevant references from included studies will be sought to retrieve additional eligible studies.

### 2.4 Data collection

#### 2.4.1 Study selection

**Figure 1:**
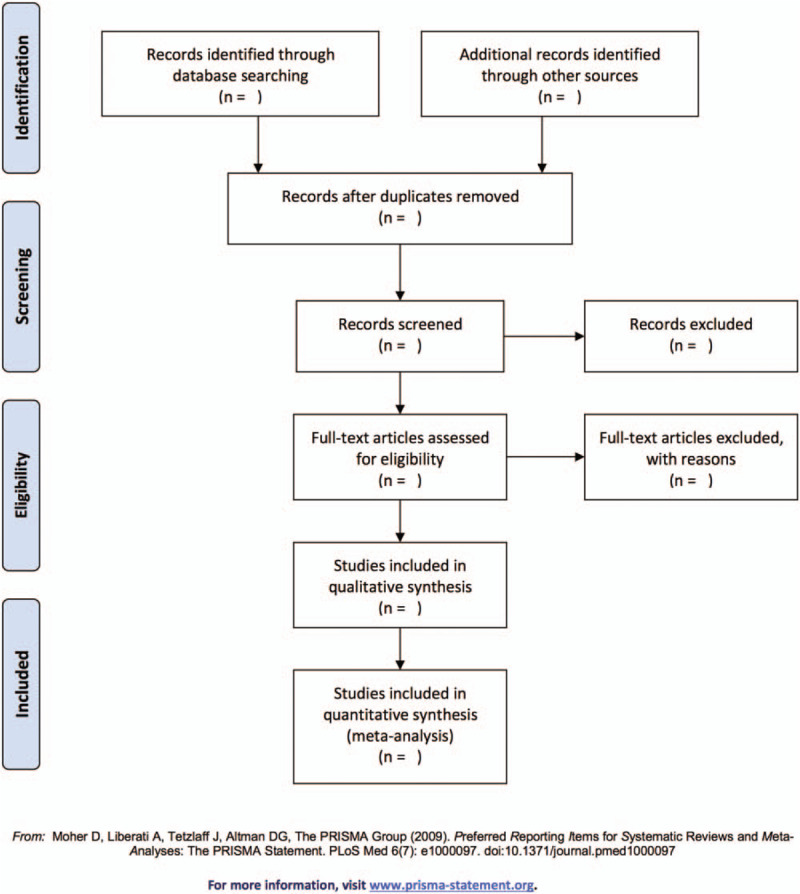
Flow diagram of study selection.

Basing on the inclusion and exclusion criteria, 2 reviewers (XS and SF) will independently review all identified data and duplicate literature will be removed. Titles, abstracts, and full-text articles will be screened and data will be extracted independently by those reviewers, with discrepancies discussed with the third reviewer (GS). If raw data are not directly provided in the text or tables, figures in the study would be referred to. Once relevant details are insufficiently reported in studies, attempts will be made to contact the study investigators for further information. The selection process will be shown in a Preferred Reporting Items for Systematic Review and Meta-analysis (PRISMA) flow chart (Fig. 1).

#### 2.4.2 Data extraction

Two reviewers (XS and SF) will perform the data extraction, and a third viewer (GS) will be involved in a discussion for any disagreements. The following information of eligible articles will be extracted to a data extraction form: the first author, published year, country of origin of the population studied, study design, sample size, health status, intervention characteristics (dosage, treatment duration), control (placebo or other) and outcome data.

### 2.5 Quality assessment

Based on the Cochrane Handbook for Systematic Reviews (version 5.3.0), we will assess the methodological quality of all studies.^[20]^ The risks of bias will be classified as low, unclear, or high by evaluating the 7 components as random sequence generation, allocation concealment, blinding of outcome assessment, blinding of participants and personnel, incomplete outcome data, selective outcome reporting, and other bias. Two independent reviewers (XS and SF) will conduct this assessment, and a third reviewer (GS) will be consulted for any disagreements.

### 2.6 Data analysis

#### 2.6.1 Measurement of the treatment effect

We will calculate the weighted mean difference and 95% confidence intervals of all outcomes: liver histology (steatosis score, hepatocellular ballooning score, lobular inflammation score, fibrosis score, and NAS), liver enzymes (ALT, aspartate transaminase, and γ-glutamyl transferase), and adverse events. If raw data are not reported, we will impute unreported means ± SDs using established methods via other information (eg, confidence intervals or median and interquartile range, etc) provided in the publication.^[21]^ If only providing data pre and post-intervention period, we will calculate the change of mean ± SDs using the formula recommended in the Cochrane Handbook Version 5.3.0.^[20]^

#### 2.6.2 Dealing with missing data

If raw data are not directly provided in the text, tables, or figures in the study will be referred to. Once relevant details are insufficiently reported in studies, authors will be contacted and the ClinicalTrials.gov register will be searched for further information.

#### 2.6.3 Assessment of heterogeneity

Study heterogeneity will be tested by *χ*2-based Cochran *Q* statistic and *I*^2^ statistic (*P* value < .10 or *I*^2^ statistic >50% indicated significant heterogeneity). The random-effects model (inverse variance method) of analysis will be used to pool the estimations of weighted mean difference across studies if significant heterogeneity is detected. In other cases, the fixed-effects model (inverse variance method) will be employed.

#### 2.6.4 Assessment of reporting biases

Using the funnel plot, Egger and Begg test to judge publication bias. In terms of accuracy, the Funnel plot is not as good as Egger test and Begg test, while Begg test is not as sensitive as Egger test. When the 3 results are inconsistent, first give up the Funnel plot. When the Egger test and the Begg test result are opposite, the result of the Egger test will be used as the result. And the trim-and-fill method will be performed to adjust for publication bias in meta-analysis.^[22]^

#### 2.6.5 Subgroups analysis and sensitivity analysis

Subgroup analysis is prespecified according to study population (children and adults), doses of supplementation (≤3 g/d and > 3 g/d), duration (≤6 months and > 6 months), and type of supplementation (DHA alone, EPA alone and the combination of EPA and DHA). Sensitivity analysis will be performed by removing a single trial each time and repeating the meta-analysis to assess the reliability and stability of the pooled results.

## 3 Discussion

NAFLD is emerging as a major health problem worldwide due to its high prevalence and the associated risk of liver-related consequences (liver cirrhosis and hepatocellular carcinoma).^[1,2,6]^ The role of n-3 PUFAs as a potential treatment for NAFLD has attracted much attention. Numerous studies have demonstrated the improvement effect of PUFAs therapy on liver fat in NAFLD patients but yielded controversial effects on other liver-related outcomes, such as liver enzymes and liver histology.^[16–18]^ In the present study, we will systematically review RCTs to evaluate the efficacy and safety of n-3 PUFAs therapy on liver-related outcomes in NAFLD patients. These findings will provide guidance to clinicians and patients on the use of n-3 PUFAs for NAFLD.

## Author contributions

**Conceptualization:** Xiao-yan Shi, Qiu Chen.

**Data analysis:** Xiao-yan Shi, Jia Yao.

**Data extraction:** Si-min Fan, Xiao-yan Shi, Guo-mei Shi.

**Funding acquisition:** Yu-guo Xia.

**Methodology:** Qiu Chen.

**Project administration:** Qiu Chen.

**Resources:** Qiu Chen.

**Software:** Guo-mei Shi, Yang Gao.

**Writing – original draft:** Xiao-yan Shi, Si-min Fan.

**Writing – review & editing:** Xiao-yan Shi, Yu-guo Xia.
